# Review of personal radiation exposure dose and history of the interventional procedure records for 40 years

**DOI:** 10.1007/s11604-021-01147-z

**Published:** 2021-06-12

**Authors:** Kimiyoshi Mizunuma

**Affiliations:** Department of the Diagnostic and Interventional Radiology, Nasu Red Cross Hospital, 1081-4, Nakadawara, Ohtawara, Tochigi 324-8686 Japan

**Keywords:** Effectiveness dose, A crystalline lens radiation exposure dose, Interventional radiology

## Abstract

**Objective:**

To inspect personal dose as an interventional radiologist for 40 years, to assess the enforcement number of interventional radiology (IR), and to check for radiation cataract.

**Materials and methods:**

I evaluated my own effective dose, an equivalent dose to the lens of the eye (EDL), and the number of IR procedures between March 2019 and June 1979. I examined the lens in June 2019 as a radiologist for 40 years.

**Results:**

The accumulation dose was 0 mSv in 1979–88. During 1989–93, the right crystalline lens equivalence of the value dose was measured. During 1993–96, two badge items for the head, neck, and abdomen were present. Both were distributed, but attaching to the same part and reversing occurred frequently. The EDL of the recent 5 years has exceeded 100 mSv. No association with the number of IR procedures was recognized. Posterior subcapsular vacuoles (PSV) as the early changes of the radiation cataract were recognized as four on the left and one on the right.

**Conclusion:**

It is important to get accustomed to film badge wearing, and the cancelation of making a mistake in the wearing part. Radiologists should check the PSV at a stage beyond a certain constant dose.

## Introduction

In 2011, the International Commission on Radiation Protection (ICRP) issued a recommendation on the equivalent dose limit for the lens of the eye for occupational exposure, which will be implemented in Japan from April 2021 [1, 2]. Along with this recommendation, I decided to verify the substance of my personal individual dose management that I have been working as a radiologist for 40 years, the relationship between equivalent dose to the lens of the eye and the number of interventional radiology procedures, and the presence or absence of radiation cataract.

## Materials and methods

I tallied my individual effective doses (ED) and equivalent doses to the lens of the eye (EDL) during June 1979–March 2019, from the data of personal dosimetry service of Chiyoda Technol Corporation. Next, I added up my enforcement interventional radiology (IR) number in the same period. If impossible, the reason was looked for. The year has been mentioned on this paper as Japanese fiscal year from April to March next year. Finally, I took the lens examination in June 2019, when I was active as a radiologist for 40 years.

## Results

### Individual radiation doses and enforcement IR number

The cumulative dose was 0 mSv during 1979–1989. As the individual dose measurement was managed by uniform exposure, it was performed by the chest film badge, which was mounted at the chest pocket of the white robe. The film badge was not mounted or placed inside the protective clothing. The IR number could not be added up in this period, because my working hospitals were Tokyo Medical University Hospital, Jikei University Hospital (JUH), Jikei University Aoto Hospital, and Ohtawara Red Cross Hospital (ORCH), which changed the name to Nasu Red Cross Hospital (NRCH) at the time of new construction move in 2012.

**Fig. 1:**
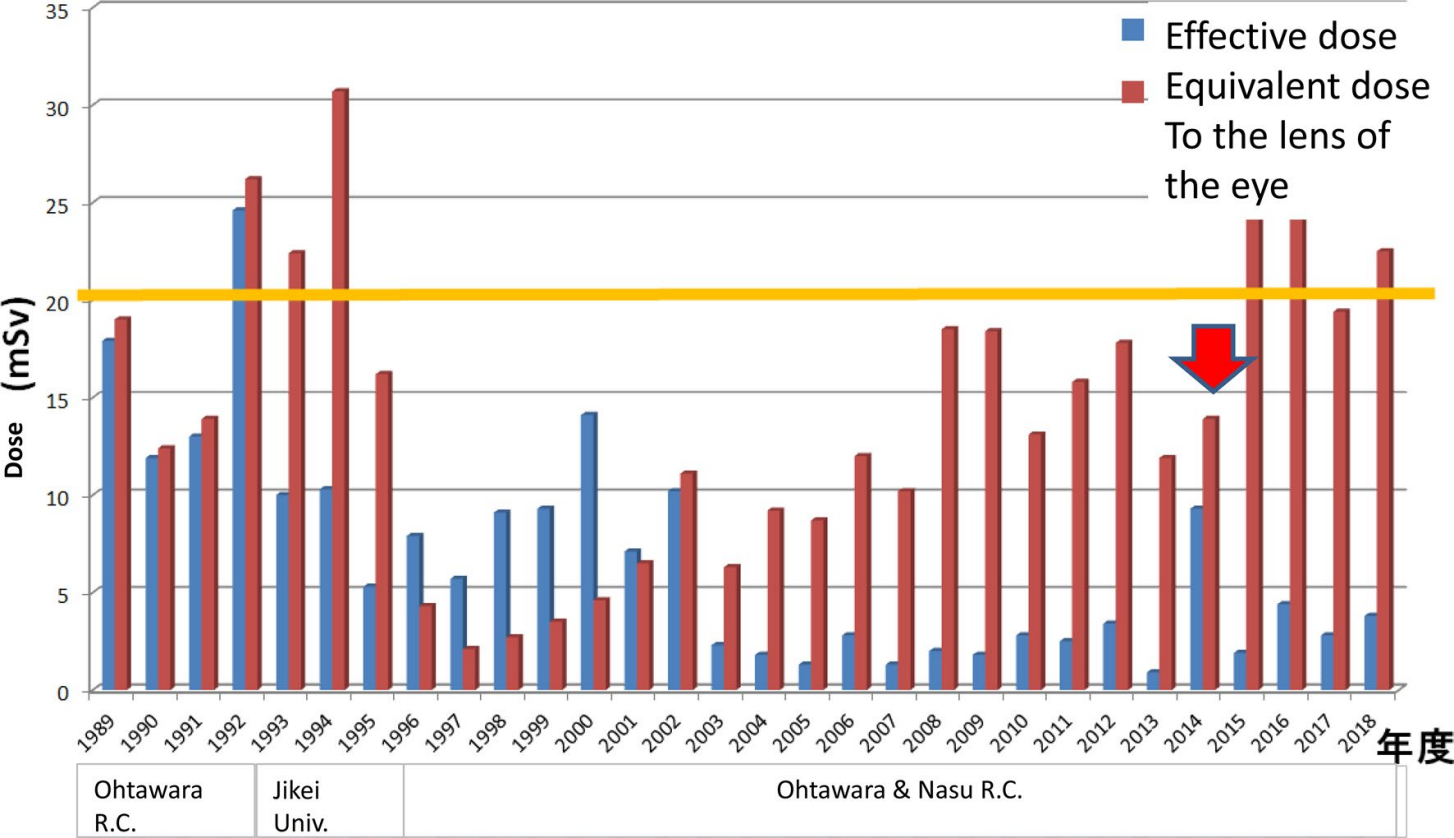
Effective dose and the equivalent dose to the lens of the eye (1989.4–2019.3) Effective dose became higher than the equivalent dose to the lens of the eye only in 2014 and I thought that there was the misunderstanding of the badge and performed a review again

Figure 1 shows the graph that expresses ED and EDL year wise from 1989 to 2018. I attached the film badge to the chest outside of protective clothes from 1989 to 1992. Two badges for the chest, head, and neck were managed in JUH from 1993 to 1995; however, they were managed only in head and neck use. I resumed work in the ORCH April 1996 onwards, and ED exceeded EDL until 2002; it is supposed that the misunderstanding of the wearing part of the badge was frequent. The measurement device turned into a glass badge from a film badge in 2003. The ratio of ED to EDL of radioactivity was abnormal in 2014. The annual dose record was reexamined.

**Fig. 2:**
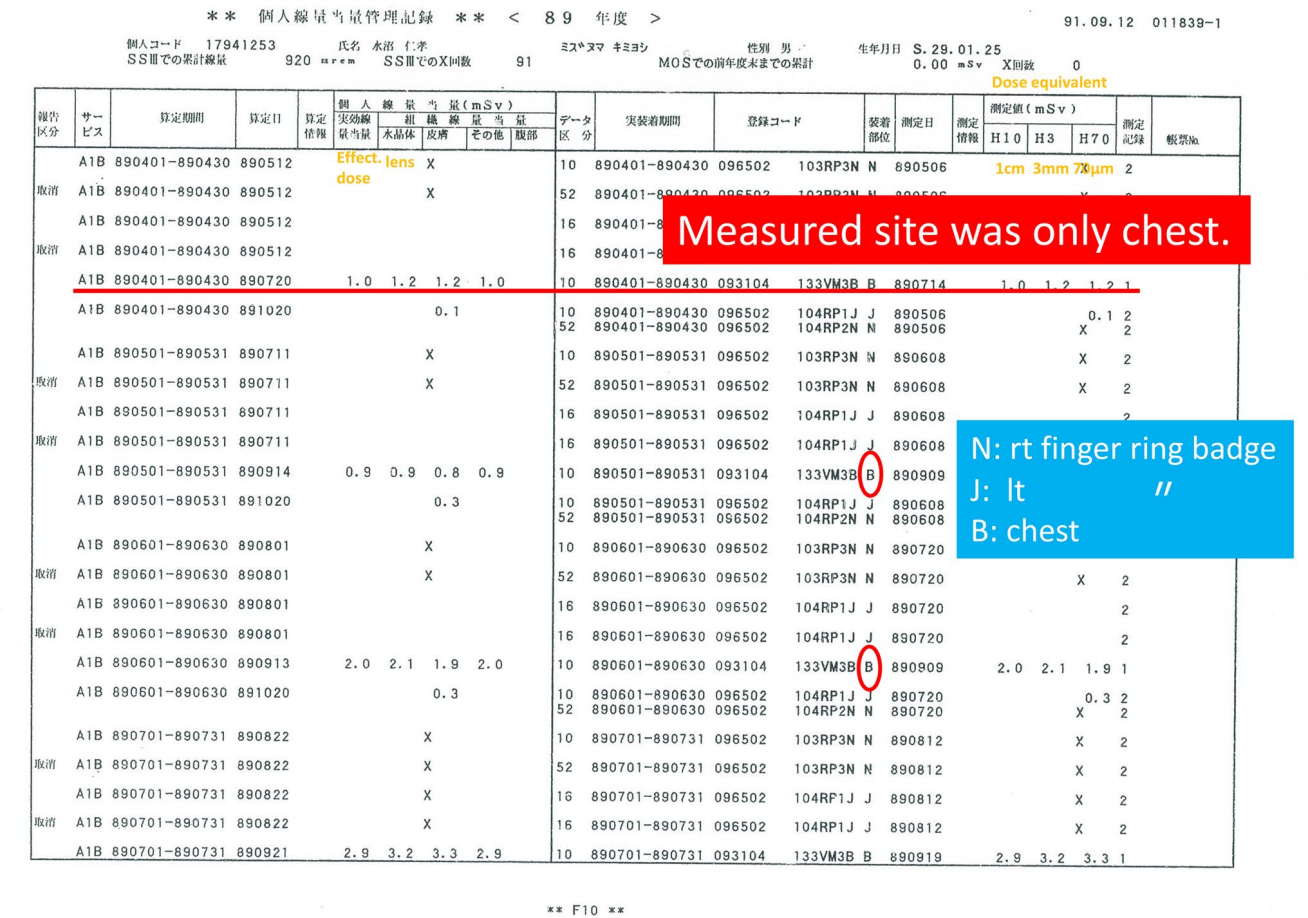
Personal equivalent dose record when I was in the ORCH until June for 1989 through 1993. In wearing part of film badge, B means for chest, N for the right finger ring badge and J for the left ring badge, but the measurement was done only at the chest

Figure 2 shows my personal equivalent dose record when I was in the ORCH until June 1989 through 1993. According to the part the film badge was worn, the representation B means for chest, N for the right finger ring badge, and J for the left finger ring badge, but the measurement was performed only at the chest. EDL (H3 mm or H70 µm) became approximately the same as ED (H10 mm) from the years 1989–1992. Since I put only the badge for chest on the outside of protective clothes, presumably the relatively right EDL was recorded. The LED recorded in 1992 exceeded 20 mSv.

**Fig. 3:**
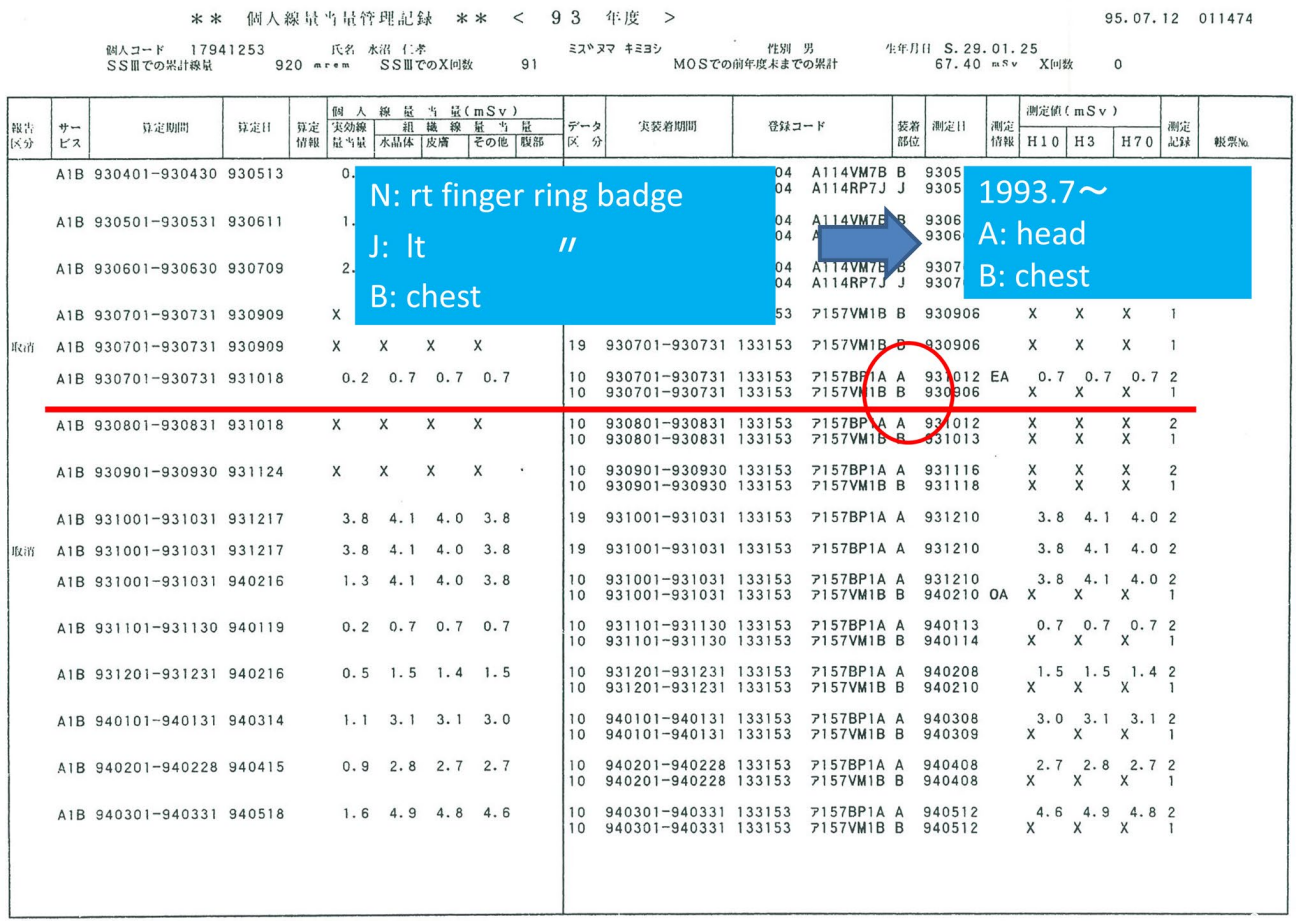
Wearing part of film badge was changed in July, 1993. **A** is badge for head and neck, and **B** is for chest. I attached (**A**) badge to the outside of the protection clothes, and it was supposed that it was not distributed because the chest use was measurements 0 mSv. Selection in the measurement ways with “uniform exposure” or “non-uniform exposure” as personal dose management, was entrusted to each facilities. It was guessed that JUH chose “uniform exposure”

From July 1993 to March 1996, my duty was at JUH. I enforced IR procedures for 3–3.5 days a week, using the imaging intensifier type digital subtraction angiography (DSA) equipment. There were two badge items of (A) for the head and neck and (B) for the chest, but I attached (A) badge to the outside of the protective clothes, and it was assumed that it was not distributed because the chest use was 0 mSv (Fig. 3). Selection in the measurement ways with “uniform exposure” or “the non-uniformed exposure” as personal dose management, was entrusted to each facility. It was assumed that JUH chose “uniform exposure” My EDL exceeded 20 mSv in 1993 and 1994. This became a problem in the Radiation Committee of the JUH because the radiographer-in-chief omitted all protection boards (face protector, side protector, skirt protector, etc.) around the DSA table, to cut down on expense, at the time of DSA introduction. The protection boards were promptly installed, and the exposure dose decreased in 1995. In April 1996, I started working in ORCH again. ED and EDL reversed from 1996 through 2001, and it seemed that the misunderstanding of the badge-wearing part was frequent. A personal dosimetry device was changed from a film badge to a glass badge in 2003. The ratio of ED to EDL of radioactivity was abnormal in 2014. By the review of the personal dose records, it was thought that there was the mistake in mounting the dosimetry devices, and they were retouched. Similar mistake was found in 2016, and it was retouched, too.

**Fig. 4:**
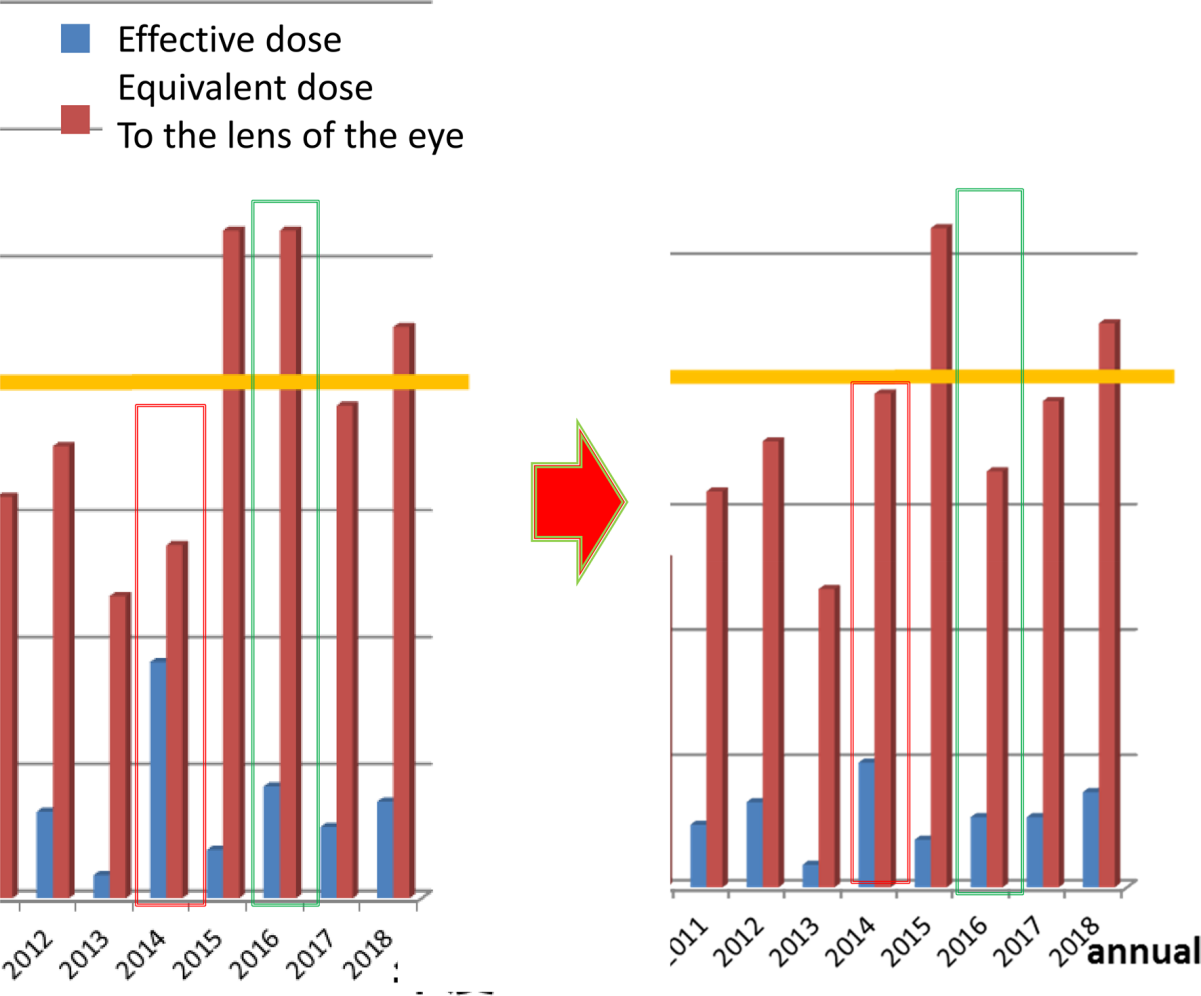
The ratio of the effective dose:ED to the equivalent dose to the lens of the eye: EDL in 2014 was abnormal. By the review of the annual dose record, the mistake in mounting the badge was thought, and they were retouched. Similar mistake was found in 2016, and it was retouched, too. EDL increased from 2008, and the totals after 2014 of 5 years came to exceed 100 mSv

EDL increased from 2008, and the total of 5 years post-2014 exceeded 100 mSv. This is mentioned above (Fig. 4).

**Fig. 5:**
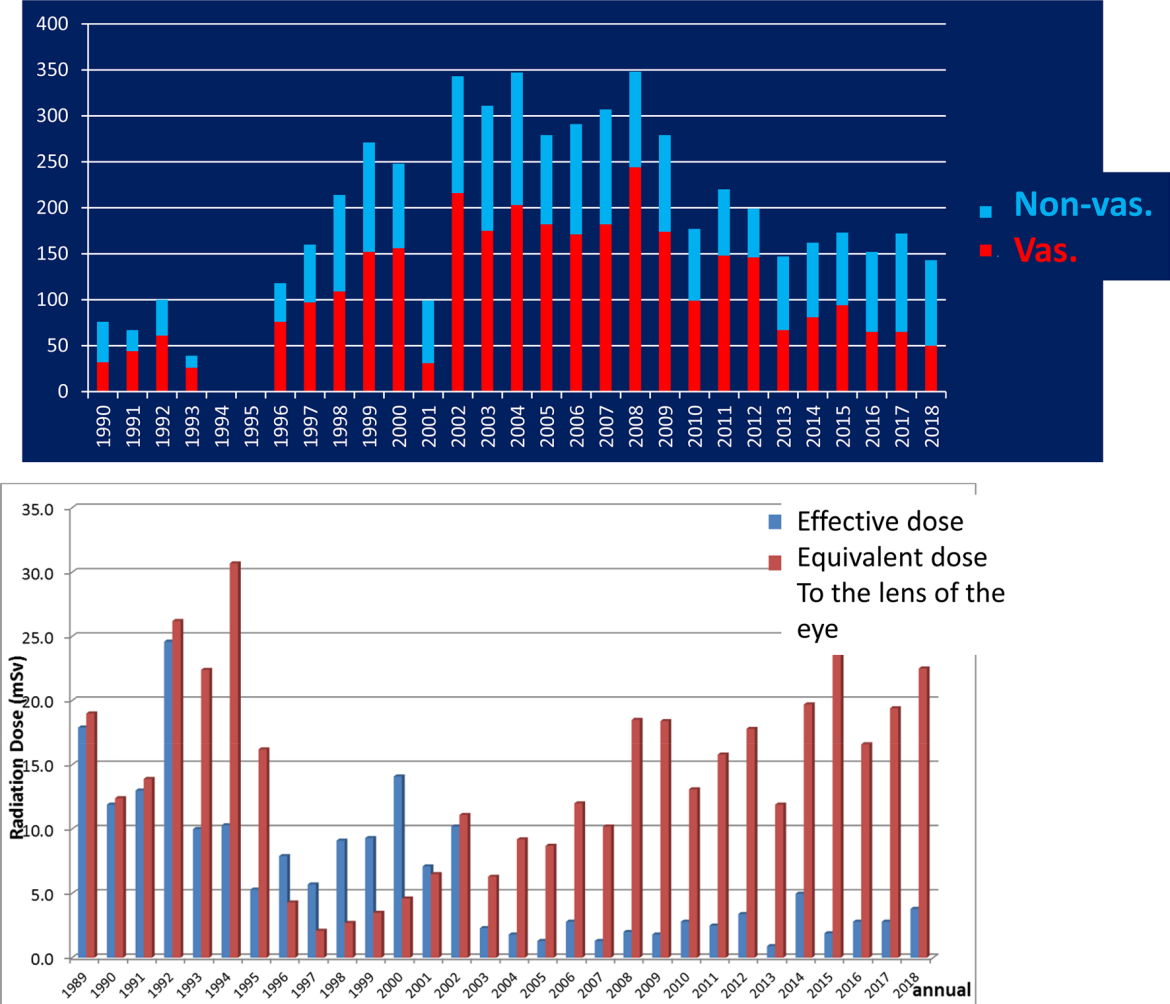
Upper line: annual number of my enforced interventional radiology from 1990 to 2018. Second line: my effective dose and equivalent dose to the lens of the eye. There was no correlation

I compared the personal exposure dose with the number of IR enforcements I performed, however, the correlation was not observed. I could not add up the number of periods when I was in the JUH from July 1993 to March 1996; it was the number of IR enforcements in the ORCH and NRCH (Fig. 5). Table 1 shows the patient exposure dose according to the maneuver of the IR that I enforced. It is the mean/median and standard deviation (SD). Vascular IR has a higher exposure dose than non-vascular IR, but it is not possible to suppose that the quantity from this number about the practiced hand radiation exposure as the non-vascular IR cannot use them unconditionally, but a phase image is not hard to resemble the things less than 1/10 generally, whereas the protection board of the table circumference functions in the case of vascular IR.

**Table 1:** Patient exposure dose according to the maneuver of the IR which I enforced

	Mean/median	SD (*mGy*)	#
Vascular IR			
TACE	1556/1320	1215	840*
Emer. TAE	1042/707	952	
CVP	12/9	11	
Non-vas. IR			
PTCD	144/58	187	664**
PTGBD	87/49	206	966**
Bil. Stent	591/137	1132	130**
Perc. Drain	104/44	172	

Left column is IR, middle is mean/median and right column is standard deviation: SD

*TACE* Transcatheter Arterial Chemo-Embolization, *Emer. TAE* Emergency Transcatheter Arterial Embolization, *CVP* the totally implantable Central Venous access Port installation, *PTCD* Percutaneous Transhepatic Cholangial Drainage, *PTGBD* Percutaneous Transhepatic GallBladder Drainage, *Bill. Stent* percutaneous Biliary Stent installation, *Perc. Drain.* Percutaneous Drainage

*2007/10–2019/12

**1983–2018

### Crystalline lens examination for radiation cataract

#### About radiation cataract

**Fig. 6:**
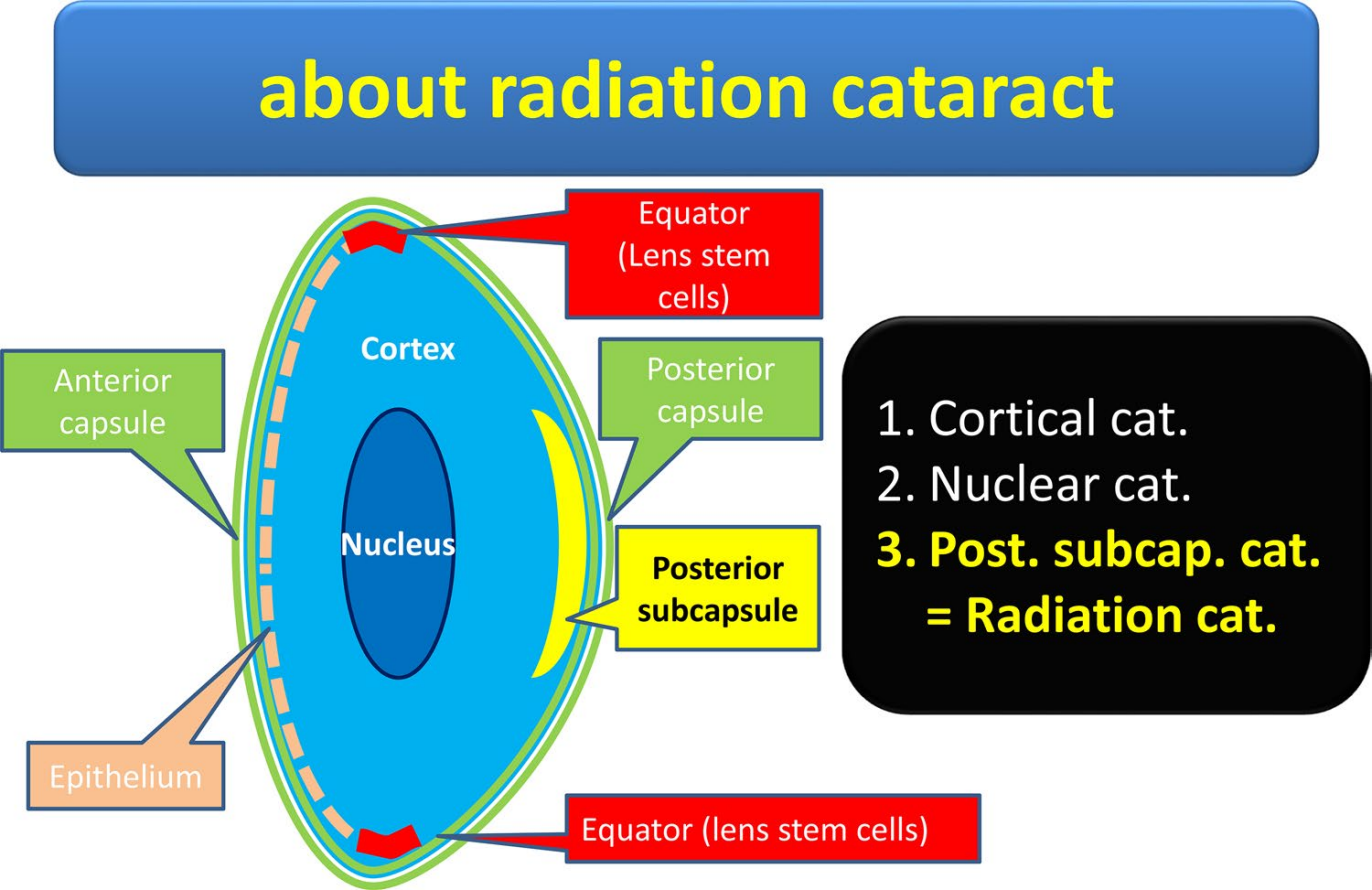
About radiation cataract. Cataracts are classified in three kinds; cortical cataract, nuclear cataract and posterior subcapsular cataract. The former two were age-related cataracts, and the last one was characteristic of radiation-induced cataract. However, a new model was suggested as outbreak mechanism of the radiation cataract by adding past documents rearranging and biological examination in 2014. It has the early-onset posterior subcapsular cataract: PSC of the threshold type and the late-onset posterior subcapsular cataract and cortex cataract of the non-threshold type. Exposure to lens stem cells at the equator, damages DNA and causes structural changes in crystalline. The degenerated cells migrate from the equator to the posterior capsule, stray into the central posterior subcapsule, and become cloudy. This is the early-onset posterior subcapsular cataract. In addition, the late-onset posterior subcapsular cataract and cortex cataract occur as a result that a radiation let aging of the crystalline lens accelerate

Cataracts include cortical cataracts, nuclear cataracts, and posterior subcapsular cataracts. The former two are age-related cataracts, and the last is a characteristic of radiation-induced cataract that causes visual deterioration [3]. However, a new model was suggested as an outbreak mechanism of radiation cataracts by adding past documents and biological examination in 2014. It has early-onset posterior subcapsular cataract (PSC) of the threshold type, late-onset posterior subcapsular cataract, and cortex cataract of the non-threshold type [4]. When the lens stem cells in the equatorial region of the lens are exposed, deoxyribonucleic acid (DNA) is damaged, causing structural changes and degeneration in crystals. Degenerated cells move from the equator to the posterior capsule side and invade the central posterior capsule, causing opacity. This is the early-onset posterior subcapsular cataract (Fig. 6). It starts with petechial turbidity and vacuoles, progresses to mottled turbidity, granular turbidity, donut-shaped turbidity, dish-shaped posterior subcapsular opacity, and finally leads to marked deterioration of visual function [5]. The Merriam–Focht scoring system and its modified method are known as diagnostic criteria for radiation cataracts [6–8] (Table 2). Fundamental criteria are based on the Stage 1 cataract, which has any one of point cloudiness > 0.25 mm in diameter (> 10 mm), vacuoles (> 5 mm), cortical spokes, water clefts, or granulated opacities. Stage 2 is the progression in the elements of Stage 1. Stages 3–5 had visual disability changes. Early changes mean in Stage 1.

**Table 2:** Merriam–Focht scoring system and its modified method

Diagnostic criteria of radiation cataract: abridged edition	
Early lens changes	
Under Stage 1	
Stage 1 cataract (onset)	
Point cloudiness more than 0.25 mm in diameter (> 10)	
Vacuoles (> 5)	
Cortical spokes	
Water clefts	
Granulated opacities	
Stage 2 cataract (progression)	
Stage 3–5 cataract (visually disabling changes)	
	Merriam–Focht scoring system and modification

Fundamental criteria is based on the Stage 1 cataract, which has any one of point cloudiness more than 0.25 mm in diameter (> 10), vacuoles (> 5), cortical spokes, water clefts or granulated opacities. Stage 2 is progression in elements of Stage 1. Stage 3 ~ 5 has visually disability changes. Early changes means under Stage 1

Late-onset posterior subcapsular cataracts and cortical cataracts occur as a result of accelerated aging of the crystalline lens.

#### Individual crystalline lens results

**Fig. 7:**
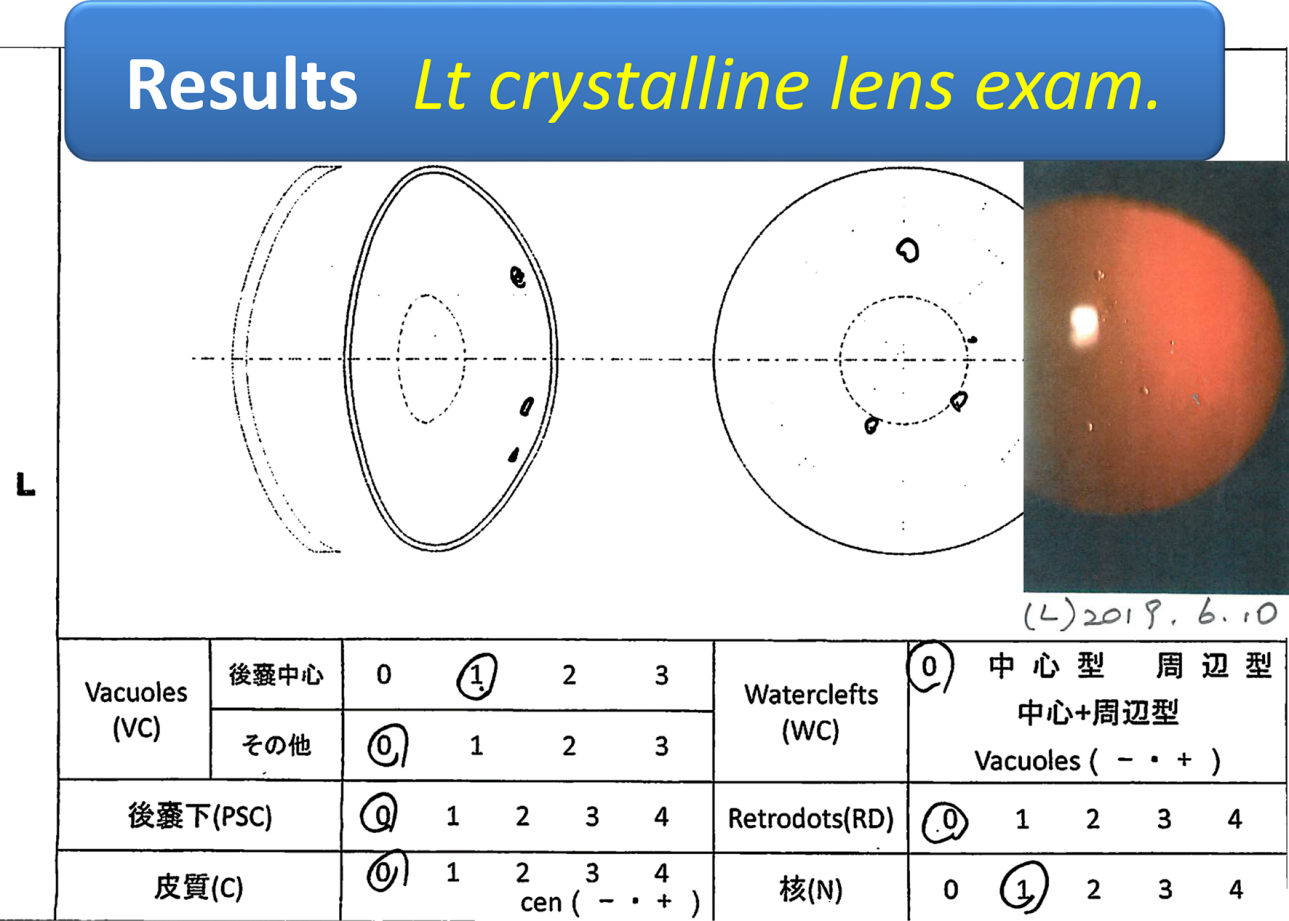
Left crystalline lens result: four vacuoles in the central posterior capsule: CPC, with mild nuclear cataract change

**Fig. 8:**
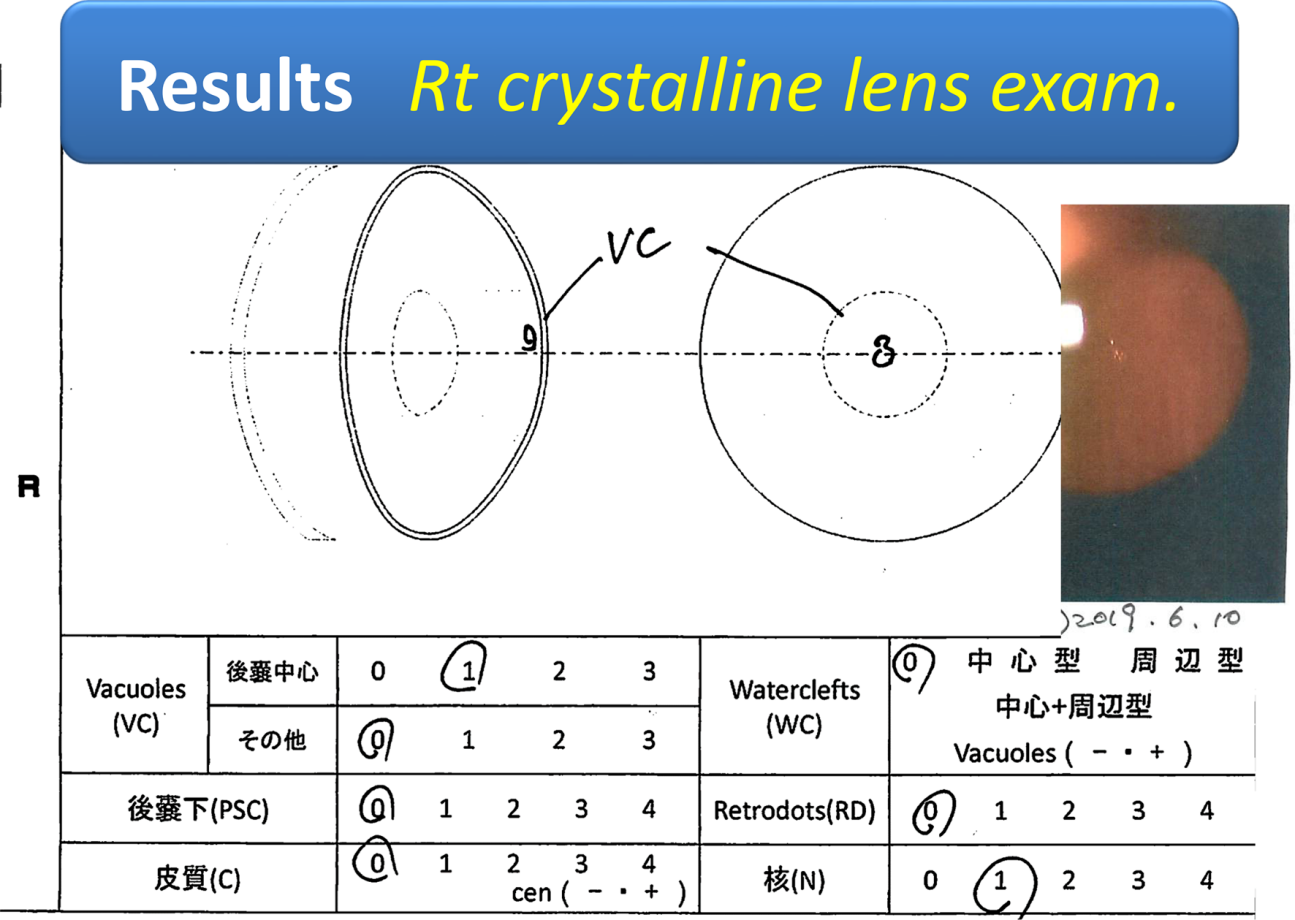
Right crystalline lens result: a vacuole in the CPC

The left lens had four vacuoles in the central posterior capsule (CPC), with mild nuclear cataract change (Fig. 7). The right lens was a vacuole in the CPC (Fig. 8). Vacuoles in both lenses were under 5, which was less than stage 1 and was equivalent to “Early lens changes.” When comparing both eyes, vacuoles were more in the left eye than in the right eye, and it was more affected by radiation exposure, which supported the conventional wisdom.

## Discussion

### Personal dosimetry

According to Chiyoda Technol’s website, the company started a personal dosimetry service in 1954. The Government of Japan enacted a regulation based on the Labor Standards Act in 1959 with the aim of preventing radiation hazards to workers engaged in work that may be exposed to radiation. With the enactment, the measurement of external and internal exposure doses of radiation workers was stipulated in chapter 2, article 8 of the Ionizing Radiation Hazard Prevention Regulations.

In this study, the dosimetry value for 10 years from 1979, when I graduated from medical school, was 0 mSv. It is probable that the initial education on individual exposure dose management was insufficient or lacking. A mistake in mounting the dosimetry device has often occurred. It is also possible that the size and shape of a couple of devices are exactly the same, and the colors of the labels are slightly different, which is another factor that causes an error. That mistake is not something that can only happen to a particular individual, but can happen to anyone [9]. This is confusing in dosimetry because it is not clear whether medical personnel involved in radiation work are dose-controlled by “effective dose” (uniform exposure) or “equivalent dose” (non-uniform exposure). This is one of the reasons for this.

### Lens exposure protection

In line with the 2011 ICRP recommendations (100 mSv in 5 years, not more than 50 mSv in a year), it exceeded 100 mSv in 5 years from 2014. It is desirable to reduce the annual lens exposure to 20 mSv or less; however, in my case, 5 years since 1989, in which the lens exposure exceeded 20 mSv were recognized. Supposedly, 2392 more than 20 mSv LED, and 426 more than 50 mSv in medical professionals [10]. Further protection against the lens is desirable.

This study also shows that the installation of protective plates around the DSA table can significantly prevent scattered radiation exposure in patients. Thus, the installation of protective plates around the DSA table is mandatory.

When the two eyes were compared, the left eye had more vacuoles than the right eye and was more affected by radiation exposure. Fortunately, my own crystal pair evaluation was under Stage 1 on a radiation cataract diagnostic standard, however, radiation cataract due to low-dose exposure progressed extremely slowly over a long period of time, and the period until cataract onset that affects visual function has been reported inversely proportional to the dose [3].

In the measurement of the backscattered light intensity using the Scheimpflug slit camera, which was conducted for the members of the Japanese Society of Interventional Radiology (JSIR) in 2006, the decrease in transparency was dominant in IVR physicians [11]. In assessing the effects of radiation on the crystalline lens, it is said that visual inspection using a slit lamp microscope and earlier subclinical crystalline lens changes (such as measurement of backscattered light intensity) should be incorporated [5]. It is considered necessary for medical personnel who are constantly exposed to medical treatment when the law is enforced to undergo such inspections at the time of entering a job and at certain intervals. Although these inspections are sung in Japanese ordinance, they are not so well known [12]. In addition, only three institutes in Japan exist where the high-end model of this inspection equipment is introduced. These are the research laboratories working on the influence of atomic bombs in Hiroshima, Nagasaki, and Chiba. Low-end model equipment was introduced to seven university hospitals indicating that it was not suitable for practical applications. For the crystalline lens equivalent radiation dosage safety limit revision for healthcare workers, nationwide system construction to detect an increase in the backscattering optical power of the posterior capsule of the crystalline lens early is expected.

## Conclusion

To prevent radiation cataract, it is necessary to first educate the proper methods of radiation exposure protection and personal dose control, and subsequently, it is desirable to enhance protective equipment. It is necessary to measure the backscattered light intensity at regular intervals, such as when entering a job, for medical workers who are constantly exposed to medical care when the law is enforced.
